# Current clinical practice in corneal crosslinking for treatment of progressive keratoconus in four Nordic countries

**DOI:** 10.1111/aos.15213

**Published:** 2022-07-10

**Authors:** Ingemar Gustafsson, André Vicente, Anders Bergström, Ulf Stenevi, Anders Ivarsen, Jesper Østergaard Hjortdal

**Affiliations:** ^1^ Department of Clinical Sciences, Department of Ophthalmology Lund University, Skåne University Hospital Lund Sweden; ^2^ Department of Ophthalmology Sahgrenska University Hospital Mölndal Sweden; ^3^ Department of Ophthalmology Aarhus University Hospital Aarhus Denmark

**Keywords:** corneal crosslinking, keratoconus, questionnaire, survey

## Abstract

**Purpose:**

To evaluate clinical practice in the diagnosis and treatment of progressive keratoconus with corneal crosslinking (CXL) in four Nordic countries.

**Methods:**

A questionnaire was sent to all centres at which keratoconus patients are evaluated and CXL is performed in Sweden, Denmark, Norway and Iceland. Nineteen of 20 centres participated.

**Results:**

CXL is performed approximately 1300 times per year in these four Nordic countries with a population of around 21.7 million (2019). In most cases, progression is evaluated using the Pentacam HR, and the maximum keratometry reading (*K*_max_) is considered the most important parameter. The most frequently used treatment protocol in Scandinavia is the 9 mW/cm^2^ epi‐off protocol, using hydroxylpropyl methylcellulose riboflavin (HPMC‐riboflavin). The participants deemed the following areas to be in most need of improvement: adaptation of the CXL protocol to individual patients (5/19), the development of effective epi‐on treatment protocols (4/19), optimal performance of CXL in thin corneas (4/19), improvement of the definition of progression (2/19), and diagnosis of the need for re‐treatment (2/19).

**Conclusions:**

We concluded that the diagnosis of progressive keratoconus and the diagnostic equipment used are similar. Treatment strategies are also similar but are suitably different to provide an interesting basis for the comparison of treatment outcomes. The high degree of participation in this survey indicates the possibility of future scientific collaboration on CXL focusing on the areas deemed to need improvement. It would also be of interest to evaluate the possibility of creating a Nordic CXL Registry. The high number of CXL treatments performed ensures sufficient statistical power to solve many questions. Such a registry could be an important contribution to evidence‐based care and would allow for longitudinal evaluation.

## 1 INTRODUCTION

Corneal crosslinking (CXL) was introduced in 2003 (Wollensak et al., 2003) with the aim of preventing the progression of keratoconus. CXL is performed by soaking the cornea with riboflavin followed by UV‐irradiation (365 nm). Riboflavin then acts as a photoreactor and induces free radicals followed by the formation of covalent crosslinks between the collagen fibrils and between the collagen fibrils and extracellular matrix (Spoerl et al., 2007). The resulting increased biomechanical strength prevents further progression of keratoconus (Sharif et al., 2018; Wollensak et al., 2004). CXL is now a widely accepted treatment to arrest the progression of keratoconus. Although CXL has been used in clinical practice for almost 2 decades, a Cochrane review in 2015 (Sykakis et al., 2015) concluded that the evidence for its efficacy in halting disease progression is very poor. This appears to have contributed to the seemingly late approval of the method by the US FDA in 2016. The main reason for granting approval was a lack of other treatment options (Jeng et al., 2016), rather than the scientifically proven efficacy of the procedure. In a more recent Cochrane review from 2021 (Ng et al., 2021b) on transepithelial versus epithelium‐off protocols, no conclusions could be drawn regarding the efficacy of either treatment. This was partially due to the lack of well‐defined indications for treatment, i.e. the definition of progressive keratoconus (Ng et al., 2021a). Furthermore, it was found that treatment protocols were not standardised, and that there was no consensus regarding the evaluation of the outcome of treatment. It was also pointed out in this review that there is a lack of randomised clinical trials and that the sample size in studies is often small. All these factors prevent the meta‐analysis of data, which could otherwise provide evidence of the efficacy of CXL.

Given the limited evidence of the efficacy of different CXL protocols and the general lack of consensus and standardisation, it was found of interest to obtain information on current clinical practice. We, therefore, invited representatives from corneal departments in four Nordic countries where CXL is performed to participate in this study by answering an online questionnaire. In this way, we obtained information from a geographic area comprising four countries with a combined population of approximately 21,7 million (Statista, n.d.) and similar public health systems with access to free CXL treatment if indicated. Apart from obtaining information on the current clinical management of progressive keratoconus and the utilisation of CXL, the purpose was to also identify areas in need of improvement.

## 2 METHODS

This study was performed in accordance with the Swedish Ethical Review Act. Centres at which CXL was performed were contacted through a national network comprising the heads of ophthalmic departments. In Denmark, Norway and Iceland, we contacted colleagues participating in the Swedish Cornea Transplant Registry to obtain information on which centres were currently performing CXL.

We identified a total of 20 centres throughout the four countries. Specialists identified as being responsible for CXL were invited to participate in the online survey. Nineteen agreed to participate and completed the online questionnaire. The questionnaire was created in a digital survey tool provided by Lund University, which protects the data by end‐to‐end user encryption and respects the General Data Protection Regulation (GDPR) as required by law. The questionnaire is located under “supplementary information”.

## 3 RESULTS

**FIGURE 1:**
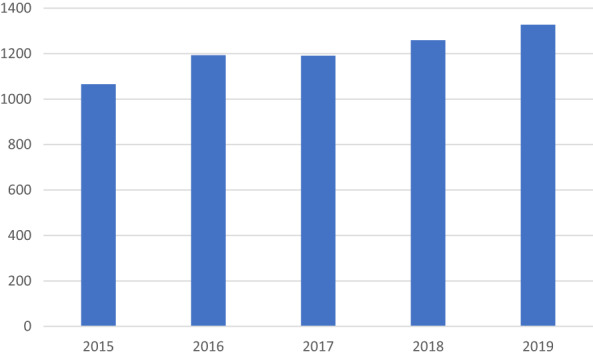
Total number of CXL treatments performed at 18 of the 19 centres.

Results are presented from 19 ophthalmology centres where CXL is currently being performed. CXL started to be performed in 2008 (median 2009, and range 2003–2018). The total number of CXL treatments performed at all the departments increased progressively from 1065 in 2015, to 1327 in 2019, as can be seen in Figure 1.

The data collected from the online questionnaire and the results are presented in Tables 1 and 2. The following instruments were used to measure progression: Scheimpflug‐based at 17 of the centres (89.5%), Placido‐based at 3 centres (15.8%), Scheimpflug‐Placido instruments at 1 centre (5.3%), and other instruments were used at 3 centres (15.8%) (Table 1).

**TABLE 1:** Clinical practice in CXL for progressive keratoconus in Scandinavia

Assessment of progression prior to CXL	*n* (%)	Topographic/tomographic measurements	*n* (%)
Adults	18 (94.7)	One measurement on each occasion	11 (57.9)
Children and adolescents (<18 years)	8 (42.1)	Mean of two or more measurements	8 (42.1)
Progression detection by number of parameters		Instrument used to define progressiom	
Combination of 2 or more?	18 (94.7)	Scheimpflug‐based	17 (89.5)
Most difficult keratoconus subgroup to diagnose		Placido‐based	3 (15.8)
Subclinical keratoconus	2 (10.5)	Schweimpflug‐Placido based	1 (5.3)
Moderate keratoconus	1 (5.3)	Other	3 (15.8)
Advanced keratoconus	12 (63.2)		
CXL protocol UVA fluence rate		CXL Protocol	
3 mW/cm^2^ (30 min)	8 (42.1)	Epi‐off	19 (100)
9 mW/cm^2^ (10 min)	12 (63.2)	Epi‐on	3 (15.8)
18 mW/cm^2^ (5 min)	3 (15.8)	Iontophoresis	0 (0)
30–45 mW/cm^2^ (16 min and 40 s)	1 (5.3)	Other	4 (21.1)
UVA irradiation		Type of riboflavin used in epi‐off protocols	
Continuous	16 (84.2)	Iso‐osmolar	7 (38.9)
Pulsed	2 (10.5)	Hypo‐osmolar	10 (55.6)
Both types	1 (5.3)	HPMC	8 (44.4)
Corneal thickness measurement		Approach with thin corneas	
After epithelial debridement	11 (57.9)	Hypo‐osmolar riboflavin	8 (42.1)
Immediately prior to UVA irradiation	12 (63.2)	Sterile water	6 (31.6)
Repeated during UVA irradiation	3 (15.8)	Other	5 (26.3)
Standard post‐CXL treatment		Complications	
Antibiotics	19 (100)	Delayed epithelial healing	14 (73.7)
Oral analgesics	17 (89.5)	Infectious keratitis	11 (57.9)
Steroids	13 (68.4)	Treatment abandoned due to pachymetry	10 (52.6)
Contact lens after treatment	9 (47.7)	Corneal melting	5 (26.3)
Topical anaesthetics	9 (47.4)	Haze warranting treatment	5 (26.3)
Topical cycloplegics	5 (26.3)	Corneal herpes infection	1 (5.3)
Non‐steroidal anti‐inflammatory drugs	4 (21.1)	Other	3 (15.8)
Other	2 (10.5)	Evaluation of the need for re‐treatment	
CXL for treatment of other diseases		Same as for untreated patients	18 (94.7)
Infectious keratitis	15 (78.9)	Other	1 (5.3)
Bullous keratopathy	2 (10.5)	Aspects in greatest need of improvement	
Other corneal ectasias	4 (21.1)	Individual adaptation of treatment protocols	5 (26.3)
After CXL		Development of effective epi‐on protocols	4 (21.1)
Follow‐up	19 (100)	Methods of performing CXL in thin corneas	4 (21.1)
Need for re‐treatment after CXL	13 (72.2)	Definition of progression	2 (10.5)
Approach if progression after treatment		Diagnosis of the need for re‐treatment	2 (10.5)
Re‐treatment with the same CXL protocol	12 (63.2)	Pain management	1 (5.3)
Re‐treatment with different CXL protocol	5 (26.3)	Awareness and earlier referral	1 (5.3)
Other	2 (10.5)		

**TABLE 2:** Parameters used for the diagnosis of progression in keratoconus

Parameter	*n* (%)
*K*_max_	17 (89.5)
Minimum corneal thickness	14 (73.7)
Medical history	14 (73.7)
Increase in astigmatism	10 (52.6)
Deterioration in BCVA	10 (52.6)
Belin ABCD Progression Display	9 (47.4)
Corneal astigmatism	8 (42.1)
Myopic change	4 (21.1)
*K*_mean_	4 (21.1)
PI (Pachymetry index)	4 (21.1)
KI (Keratoconus index)	4 (21.1)
KPI (Keratoconus progression index)	4 (21.1)
Posterior radius (r‐min)	3 (15.8)
Change in spherical equivalence	3 (15.8)
Deterioration in uncorrected visual acuity	2 (10.5)
ISV (Index of surface variance)	2 (10.5)
D‐index	2 (10.5)
IHA (Index for height asymmetry)	1 (5.3)
Other	5 (26.3)
Most important parameter in detection	
*K*_max_	11 (57.9)
Belin ABCD progression display	4 (22.1)
Increase in astigmatism	1 (5.3)
Other	2 (10.5)

Progressive keratoconus is usually an indication of CXL in adults. Progression was assessed in some way prior to CXL in adults at 18 of the 19 centres. At the centre where progression was not assessed prior to CLX treatment, all patients younger than 32 years were treated without any hard evidence for progression. Documentation of progression was not required in children (<18 years) prior to CXL treatment at the majority of the centres (*n* = 11).

Progression was defined as a change in two or more parameters at 18 of the 19 centres. The parameters used to detect progression are shown in Table 2: maximum keratometry (*K*_max_) as measured by Scheimpflug keratometry or OCT was the parameter most used (*n* = 17), followed by minimum corneal thickness (MCT; *n* = 14), medical history (*n* = 14), increased astigmatism (*n* = 10), deterioration in best corrected visual acuity (BCVA; *n* = 10), Belin ABCD Progression Display (*n* = 9) and other parameters as described in Table 2.

The magnitude of change in the parameters regarded as indication of progression varied and included: increase in *K*_max_ of 1.5 D, 1.0 D over 1 year, 0.5 D over 6 months; reduction in MCT of 30 μm, or 20 μm; and increased topographic astigmatism equal to or greater than 1.0 D. *K*_max_ was considered the most important parameter in diagnosing the progression of keratoconus prior to CXL at 11 of the centres (57.9%), followed by the Belin ABCD Progression Display at 4 (21,1%), other parameters at 2 (10.5%), and increased astigmatism at 1 (5.3%).

Topographic or tomographic measurements between clinical visits are usually compared to assess progression. A single measurement on one occasion was compared with a single measurement on another occasion at 11 of the 19 centres, while the mean of two or more measurements on one occasion was compared with the mean of two or more measurements on another occasion at 8 centres. Advanced keratoconus was considered to be the keratoconus subgroup in which it is more difficult to diagnose progression at 12 of the 19 centres, followed by subclinical keratoconus at two centres and moderate keratoconus at one centre.

The fluence rate used in the CXL treatment protocol was 9 mW/cm^2^ at 12 of the centres, 3 mW/cm^2^ at 8 of the centres, 18 mW/cm^2^ at 3 of the centres, while 30–45 mW/cm^2^ was used at one centre. Continuous UVA irradiation was used at 16 of the centres (84.2%), pulsed at two (10.5%) and both types at one centre (5.3%). The epi‐off CXL treatment protocol (with epithelial removal) was performed at all 19 centres, whereas the epi‐on CXL treatment protocol (without epithelial removal) was performed at 3 centres (15.8%); iontophoresis CXL treatment was not performed at any of the centres included in this study. Other protocols were also performed at 4 of the centres (21.1%) and included epi‐off CXL treatment protocols assisted with contact lens, epi‐off with transepithelial Phototherapeutic Keratectomy (PTK) for epithelium removal and epi‐on CXL treatment protocols with supplementary oxygen.

Hypo‐osmolar riboflavin (without dextran) was the kind of riboflavin used most frequently in epi‐off techniques at 10 (55.6%) of the centres, followed by hydroxylpropyl methylcellulose riboflavin (HPMC‐riboflavin) at 8 (44.4%) and iso‐osmolar riboflavin (with dextran) at 7 (38.9%) of the centres. Corneal thickness was measured after epithelial debridement at 11 (57.9%) of the centres, immediately prior to UVA irradiation at 12 (63.2%), and repeatedly during UVA irradiation at only 3 (15.8%) of the centres. Hypo‐osmolar riboflavin was added if the cornea was too thin to be treated safely at 8 centres (42.1%), sterile water at 6 (31.6%), while other approaches were used at 5 (26.3%) of the centres. Insufficient corneal thickness had caused the termination of a CXL procedure at least once in the past, at 10 (52.6%) of the centres.

A soft contact lens was inserted after treatment at 9 (47.4%) of the centres. Standard pharmacological treatment after CXL included antibiotics at all centres, oral analgesics at 17 (89.5%), steroids at 13 (68.4%), topical anaesthetics at 9 (47.4%), topical cycloplegics at 5 (26.3%), non‐steroidal anti‐inflammatory drugs (NSAIDs) at 4 (21.1%) and other treatments at 2 of the centres (10.5%).

Delayed epithelial healing was the most common complication reported after CXL treatment at 14 of the 19 centres, followed by infectious keratitis at 11 centres, corneal melting at 5 centres, haze warranting treatment at 5 centres, corneal herpes at one centre and other complications at 3 centres. Patients were followed up after CXL treatment at all the centres. The need for re‐treatment with CXL was identified at least once in 13 of the centres performing CXL. In cases of progression after CXL treatment, re‐treatment with the same CXL protocol was the approach of choice at 12 of the centres, re‐treatment with a different CXL protocol at 5 and other approaches at 2. The need for re‐treatment was evaluated with the same method as for untreated patients at all but one of the centres. CXL was also used for the treatment of infectious keratitis at 15 (78.9%) of the centres, bullous keratopathy at 2 (10.5%) and other indications which included pellucid marginal degeneration and other corneal ectasias at 4 (21.1%) of the centres.

Individual adaptation of the treatment protocol was considered to be the aspect in greatest need of improvement in CXL at 5 (26.3%) of the centres, followed by the development of effective epi‐on protocols at 4 (21.1%), methods of performing CXL in thin corneas at 4 (21.1%), the definition of progression at 2 (10.5%), diagnosis of the need for re‐treatment at 2 (10.5%), pain management at one (5.3%) and the need for greater awareness and earlier referral to CXL at one (5.3%).

## 4 DISCUSSION

This survey provides detailed information on current practices regarding CXL and the number of times it is performed per year in four Nordic countries. A major strength of the current study is the high rate of participation, 19 of 20 centres (95%) where CXL is performed in these four Nordic countries. Heterogeneity in the management of progressive keratoconus can be expected due to the lack of international guidelines and hard evidence for different treatment protocols. Therefore, a high participation rate is of importance to provide an accurate description of current practice patterns.

The number of CXL treatments performed during the period 2015–2019 was about 1200 per year, with a slightly increasing tendency. The population of the four Nordic countries included in this study in 2019 was approximately 21,7 million (Statista). Thus, about 6–7 CXL treatments were performed per 100 000 inhabitants per year, during this period. No epidemiological data are available on keratoconus covering all the included countries; however, data from Norway (Kristianslund et al., 2021) suggested an annual incidence rate of 19.8 cases in 100 000 inhabitants and data from Denmark (Bak‐Nielsen et al., 2019) suggested an incidence rate of 3.6 cases per 100 000 inhabitants. As the number of diagnosed cases increases with time (Bak‐Nielsen et al., 2019), the difference in the incidence rate between the investigations could be explained by the more recent data in the Norwegian study. As there are no data from all included countries in this investigation, no further analysis regarding the number of CXL performed in relation to the incidence and prevalence of keratoconus could be made. However, it would be of interest to evaluate how many patients are diagnosed with keratoconus per year and the characteristics of those who are ultimately referred for CXL. In general, a diagnosis of progression is warranted in patients aged ≥18 years prior to referral to CXL. However, most clinics refer subjects <18 years of age directly to CXL upon the diagnosis of keratoconus, due to the risk of progression (Chatzis & Hafezi, 2012). The results of a recent randomised control trial suggested that 43% of children and adolescents aged 10–16 years progressed within 18 months of diagnosis, and that the adjusted odds ratio of progression was reduced by 90% if the subjects were referred to CXL upon diagnosis (Larkin et al., 2021). This underlines the importance of CXL in halting the progression of keratoconus but implied 57% overtreatment during the 18‐month duration of the study. The true degree of overtreatment should, however, be analysed in a longitudinal study over several years, as it is not known which patients will eventually progress. Such knowledge would be of considerable importance in suggesting the optimal treatment strategy for children and adolescents. However, this would require a clear definition of progressive keratoconus and longitudinal evaluation, preferably using data from a national or international registry.

*K*_max_ is the most frequently (90%) used parameter to detect progression, which other authors also have reported (Ng et al., 2021a; Sykakis et al., 2015), followed by the Belin ABCD Progression Display (Belin et al., 2017; Duncan et al., 2016). The major discrepancy between scientific and clinical practice is that corneal thickness measurements and the ABCD Progression Display are more commonly used in clinical practice. Almost all the participants (18/19) stated that progression was more difficult to assess in advanced keratoconus due to the considerable variation between measurements. This is explained by the several‐fold difference in the repeatability of measurements between low‐grade and high‐grade keratoconus, as there is an association between the measurement error and disease magnitude (Gustafsson et al., 2020). The commonly used cut‐off of a 1.0 D increase in *K*_max_ will lead to underdiagnosis of those with less advanced keratoconus and overdiagnosis of those with moderate to advanced disease. Stratified detection limits are thus warranted. “Improving the definition of progression” and “methods of diagnosing the need for re‐treatment” were the third most important aspects of CXL that need to be improved according to the participants (2/19 votes each).

The original dextran‐based iso‐osmolar riboflavin (Wollensak et al., 2003) is rarely used in the included Nordic countries, which is in contrast to most other clinical investigations where dextran‐based iso‐osmolar riboflavin is the preferred form of riboflavin or reference riboflavin (Ng et al., 2021a; Sykakis et al., 2015). The major drawback of dextrane‐based iso‐osmolar riboflavin is the thinning effect it has on the cornea due to an oncotic effect (Wollensak & Spörl, 2019). In fact, only one centre used dextran‐based iso‐osmolar riboflavin as the only form of riboflavin. Hypo‐osmolar riboflavin is more commonly used, either as the only form of riboflavin or as an additive to dextran‐based iso‐osmolar riboflavin if the cornea is very thin (9/19). However, only a few studies have evaluated the pre‐clinical (Wollensak & Spörl, 2019) and clinical (Raiskup & Spoerl, 2011) efficacy and safety of hypo‐osmolar riboflavin. Thus, more prospective investigations are required to ascertain the evidence. HPMC‐based iso‐osmolar riboflavin does not appear to cause corneal thinning (Sherif et al., 2016) and long‐term follow‐up suggests its efficacy in halting disease progression (Mazzotta et al., 2021). This, in combination with a shorter imbibition phase, could explain why it is the most popular form of riboflavin among the participants (9/19 participants).

Half of the participants (10/19) reported that they at occasions had had to abandon CXL due to insufficient corneal thickness, referring to the commonly used minimum thickness at 400 μm (Spoerl et al., 2007). However, this safety‐limit has recently been suggested to be exaggeratively conservative when using dextran‐based iso‐osmolar riboflavin (Seiler et al., 2019) which is consistent with the few reports of endothelial failure after CXL (Gokhale, 2011; Kymionis et al., 2012). According to the Beer–Lambert law, the irradiance can be reduced by increasing the corneal thickness, increasing the absorption coefficient or reducing the surface fluence rate. In clinical practice, this problem was mainly overcome by increasing the corneal thickness by the addition of hypo‐osmolar riboflavin (Hafezi et al., 2009) (8/19), sterile water (Gustafsson et al., 2017) (6/19), inserting a UV‐permeable contact lens (Jacob et al., 2014) (2/19) or by reducing the fluence rate (Hafezi et al., 2021) (2/19). Importantly, the addition of hypo‐osmolar riboflavin increases the corneal thickness which reduces the irradiance at the level of the endothelium but its lower absorption coefficient (compared to iso‐osmolar riboflavin) increases the irradiance at the level of the endothelium (Wollensak et al., 2010). As the safety‐limit strongly influences the choice of CXL‐treatment, efforts should be made to re‐define the optimal safety‐limit for different riboflavin compositions. “How to perform CXL on thin corneae” and “Developing efficacious epi‐on treatment protocols” were deemed the second most important aspects of CXL needing improvement (4/19). Epi‐on protocols were, however, seldom used. The riboflavin molecule is too large to pass through the epithelium (Huang et al., 1989) and so‐called “enhancers” must be added in order to disintegrate the epithelium (Chen et al., 2015). Alternatively, the riboflavin can be delivered through an iontophoresis‐assisted transport (Lombardo et al., 2017). However, the riboflavin concentration in the stroma remains low compared to epi‐off procedures (Franch et al., 2015; Hayes et al., 2016). This reduced intrastromal concentration of riboflavin in combination with a lower stromal oxygen concentration (Freeman, 1972; Kling et al., 2015; Richoz et al., 2013; Seiler et al., 2021) and partial UV‐reflection (Podskochy, 2004) will reduce the stiffening effect of CXL. This reduction is equivalent to 20% of that provided by the original epi‐off 3 mW/cm^2^ protocol (Wollensak & Iomdina, 2009). The clinical outcome of epi‐on protocols has been reported to be disappointing (Caporossi et al., 2013; Kobashi et al., 2018; Nath et al., 2020), although they are regarded as safer (Nath et al., 2020).

The most common fluence rate was 9 mW/cm^2^. Although this induces less stiffening than the reference 3 mW/cm^2^ fluence rate (Hammer et al., 2014), several studies have shown its efficacy in halting the progression of keratoconus (Kobashi & Tsubota, 2020; Mazzotta et al., 2021). CXL has been shown to be oxygen‐dependent, and a higher fluence rate depletes the oxygen more rapidly, reducing the crosslinking process. However, there is sufficient oxygen concentration at a depth of 200 μm, where most crosslinking takes place, in both the 3 and 9 mW/cm^2^ protocols. Of the clinics that use more than one fluence rate (3–18 mW/cm^2^), patients <18 years old are commonly treated with the 3 mW/cm^2^ protocol due to their increased risk of progression. As both “under‐crosslinking” and “over‐crosslinking” may occur (Seiler, 2021), one of the challenges lies in choosing the optimal treatment strategy for each patient to avoid continued progression post‐CXL and side effects in terms of excessive flattening. In fact, “Adaptation of the treatment protocol to individual patients” was the aspect of CXL considered to be in most need of improvement (5/19). Due to the lack of evidence on “individualised” treatment options, more efforts should be made to investigate this further. However, such investigations would depend on an accepted classification of keratoconus disease characteristics and a longitudinal evaluation.

The most serious post‐CXL complications are corneal melting and infectious keratitis. While corneal melting is limited to a few case reports, infectious keratitis appears to be more common, although the incidence differs substantially. Reported values of incidence range from 0.0017% (Shetty et al., 2014) to 1.3% (Kato et al., 2021) and 2.85% (Tzamalis et al., 2019). Soft contact lenses and steroids are commonly used in the post‐CXL treatment regime, both of which could be risk factors for infectious keratitis (Tzamalis et al., 2019). The optimal post‐CXL treatment strategy could be determined based on an evaluation of risk factors for the development of keratitis, preferably using data from a registry, as several hundred of patients must be recruited. Follow‐up of patients after CXL showed that 14 of 19 centres had experienced cases of re‐treatment. It would be valuable to determine the duration of the clinical effect of CXL in halting the progression of keratoconus to optimise follow‐up protocols. Little is known on the natural turnover of collagen (Paik et al., 2018). The turnover of collagen will reduce the degree of crosslinking and associated stiffening, although the cornea becomes stiffer with age.

In conclusion, this survey provides detailed information on the current practice regarding CXL in the four Nordic countries. Valuable scientific contributions could be obtained by extended collaboration between countries, taking advantage of the relatively high volume of CXLs performed. A Nordic registry for corneal crosslinking could provide a source of data on evidence‐based practice and allow for future longitudinal studies.

## FUNDING INFORMATION

The Foundation for the Visually Impaired in the County of Malmöhus, Sweden. The Synoptik Foundation, Denmark. The sponsors or funding organisations had no role in the design or conduct of this research.

## Supporting information


Appendix S1
