# Supplementary material for: Current clinical practice in corneal crosslinking for treatment of progressive keratoconus in four Nordic countries
Source: Acta Ophthalmol. 2022 Jul 10;101(1):109–16. doi: 10.1111/aos.15213 (PMC10084360; doi:10.1111/aos.15213)
Supplement: Supplementary file 1 — Appendix S1 [file AOS-101-109-s001.pdf]

CXL in Scandinavia NV

Respondents: 20
Answer Count: 19
Answer Frequency: 95.00%

How many CXL treatments for progressive keratoconus did did your clinic perform for each year?

2015

2015	Number of Responses
0 - 24	3 (17.6%)
25 - 49	6 (35.3%)
50 - 74	4 (23.5%)
75 - 99	1 (5.9%)
100 - 124	1 (5.9%)
125 - 149	1 (5.9%)
150 - 174	0 (0.0%)
175 - 199	0 (0.0%)
200 - 224	0 (0.0%)
225 - 249	1 (5.9%)
Total	17 (100.0%)

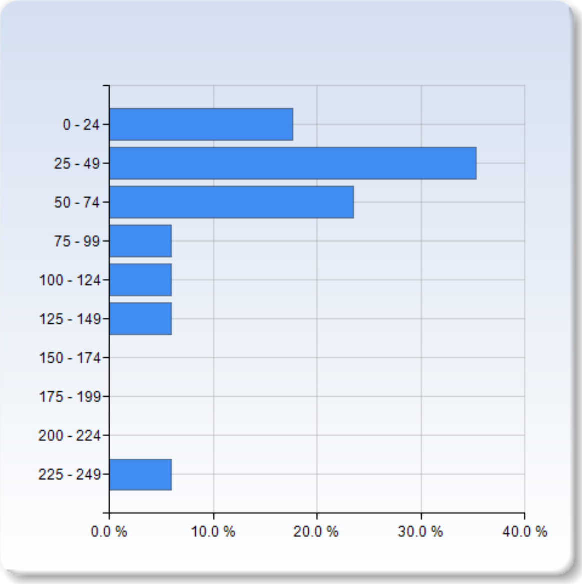

	Mean	Standard Deviation
2015	62.6	58.1

2016

2016	Number of Responses
6 - 31	3 (17.6%)
32 - 57	7 (41.2%)
58 - 83	2 (11.8%)
84 - 109	1 (5.9%)
110 - 135	2 (11.8%)
136 - 161	1 (5.9%)
162 - 187	0 (0.0%)
188 - 213	0 (0.0%)
214 - 239	0 (0.0%)
240 - 265	1 (5.9%)
Total	17 (100.0%)

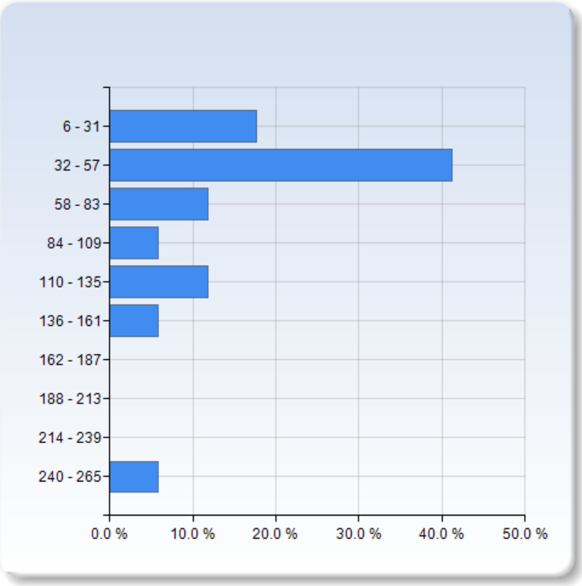

	Mean	Standard Deviation
2016	70.2	62.3

2017

2017	Number of Responses
6 - 32	7 (41.2%)
33 - 59	4 (23.5%)
60 - 86	0 (0.0%)
87 - 113	2 (11.8%)
114 - 140	2 (11.8%)
141 - 167	1 (5.9%)
168 - 194	0 (0.0%)
195 - 221	0 (0.0%)
222 - 248	0 (0.0%)
249 - 275	1 (5.9%)
Total	17 (100.0%)

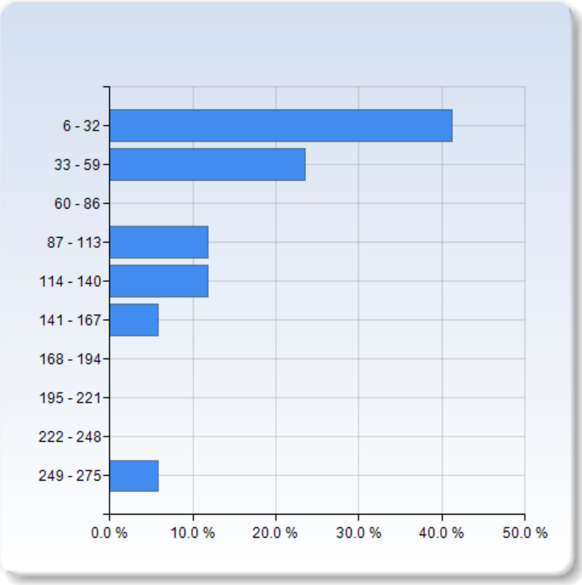

	Mean	Standard Deviation
2017	70.1	68.5

2018

2018	Number of Responses
1 - 31	6 (33.3%)
32 - 62	3 (16.7%)
63 - 93	5 (27.8%)
94 - 124	2 (11.1%)
125 - 155	0 (0.0%)
156 - 186	1 (5.6%)
187 - 217	0 (0.0%)
218 - 248	0 (0.0%)
249 - 279	0 (0.0%)
280 - 310	1 (5.6%)
Total	18 (100.0%)

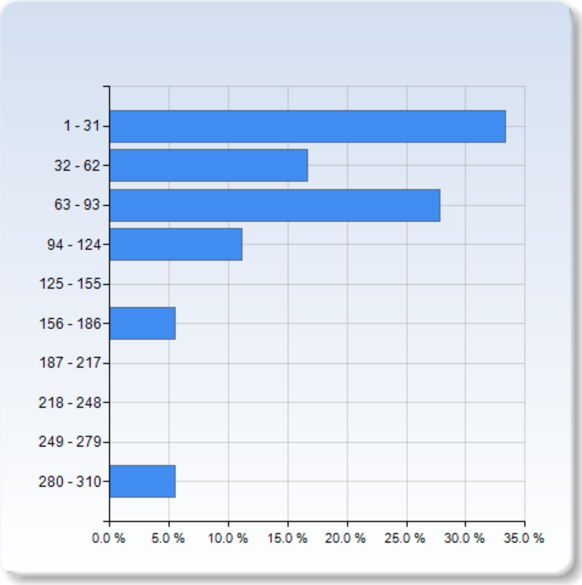

	Mean	Standard Deviation
2018	69.9	72.7

2019

2019	Number of Responses
9 - 38	7 (38.9%)
39 - 68	3 (16.7%)
69 - 98	5 (27.8%)
99 - 128	1 (5.6%)
129 - 158	0 (0.0%)
159 - 188	1 (5.6%)
189 - 218	0 (0.0%)
219 - 248	0 (0.0%)
249 - 278	0 (0.0%)
279 - 308	1 (5.6%)
Total	18 (100.0%)

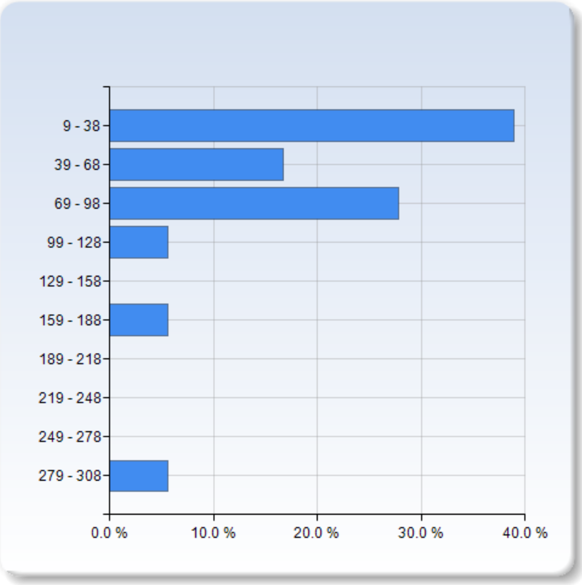

	Mean	Standard Deviation
2019	73.7	69.3

The indication for CXL is usually *progressive* keratoconus. Do you assess progression prior to CXL in adults (>18 yrs)? If not, please specify why.

The indication for CXL is usually *progressive* keratoconus. Do you assess progression prior to CXL in **adults** (>18 yrs)? If not, please specify why.	Number of Responses
Yes	16 (84.2%)
No	3 (15.8%)
Total	19 (100.0%)

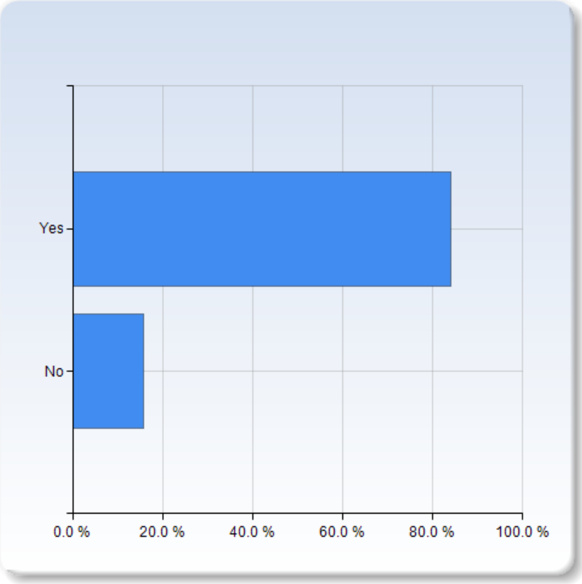

	Mean	Standard Deviation
The indication for CXL is usually *progressive* keratoconus. Do you assess progression prior to CXL in **adults** (>18 yrs)? If not, please specify why.	1.2	0.4

In general, the indication for CXL is progressive keratoconus. Do you assess progression prior to CXL in children and adolescents (<18 yrs)? If not, please specify.

In general, the indication for CXL is progressive keratoconus. Do you assess progression prior to CXL in **children and adolescents** (<18 yrs)? If not, please specify.	Number of Responses
Yes	8 (42.1%)
No	11 (57.9%)
Total	19 (100.0%)

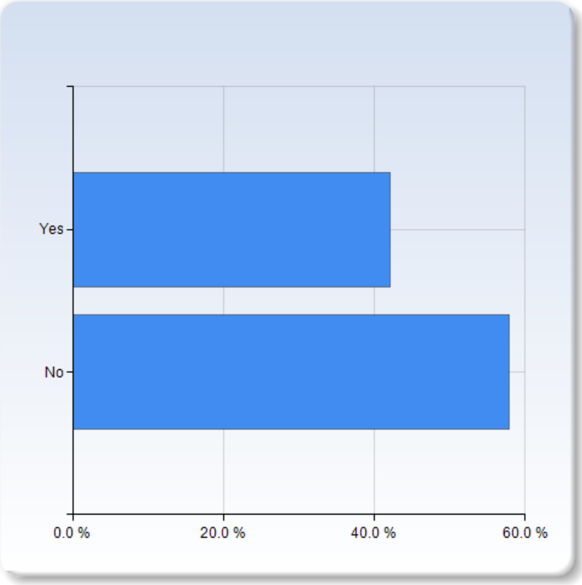

	Mean	Standard Deviation
In general, the indication for CXL is progressive keratoconus. Do you assess progression prior to CXL in **children and adolescents** (<18 yrs)? If not, please specify.	1.6	0.5

Is one parameter enough to detect progression or must it be a combination of two or more parameters?

Is one parameter enough to detect progression or must it be a combination of two or more parameters?	Number of Responses
One parameter	1 (5.3%)
Combination	18 (94.7%)
Total	19 (100.0%)

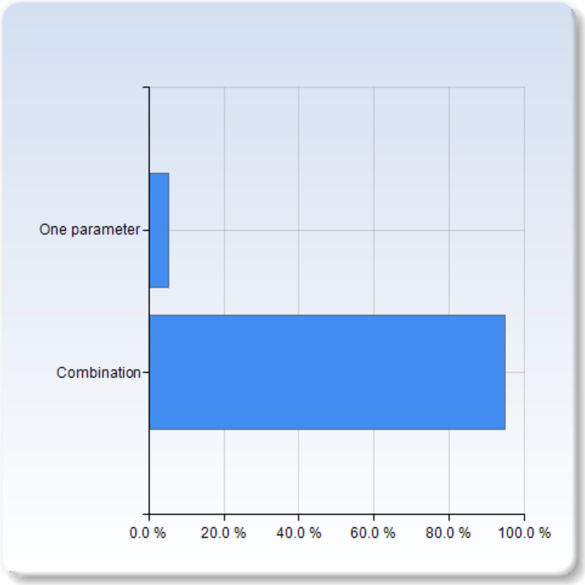

	Mean	Standard Deviation
Is one parameter enough to detect progression or must it be a combination of two or more parameters?	1.9	0.2

Mark the parameters that you use to detect progression. Please specify what change in magnitude in the comments.

Mark the parameters that you use to detect progression. Please specify what change in magnitude in the comments.	Number of Responses
Anamnesis	14 (73.7%)
Myopic change	4 (21.1%)
Change in spherical equivalence	3 (15.8%)
Increased astigmatism	10 (52.6%)
Deteriorated best corrected visual acuity	10 (52.6%)
Deteriorated uncorrected visual acuity	2 (10.5%)
Corneal astigmatism	8 (42.1%)
Kmean	4 (21.1%)
Kmax	17 (89.5%)
MCT (minimum corneal thickness)	14 (73.7%)
Posterior radius (r-min)	3 (15.8%)
ISV (index of surface variance)	2 (10.5%)
D-index	2 (10.5%)
RPI (Pachymetry index)	4 (21.1%)
ART (Ambrósio Relation Thickness)	0 (0.0%)
KI (Keratoconus index)	4 (21.1%)
KPI (Keratoconus progression index)	4 (21.1%)
IHA (Index for height asymmetry)	1 (5.3%)
"DUCK SCORE"	0 (0.0%)
Belin ABCD progression display	9 (47.4%)
Other, please specify	5 (26.3%)
Total	120 (631.6%)

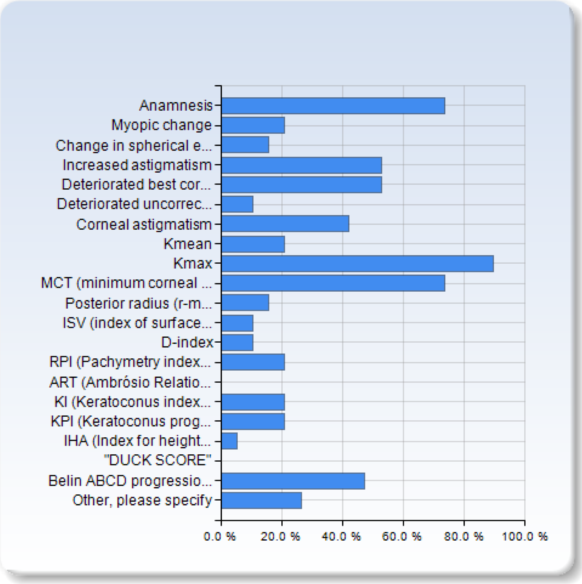

	Mean	Standard Deviation
Mark the parameters that you use to detect progression. Please specify what change in magnitude in the comments.	9.1	5.9

Indicate which parameter you believe to be the most important in detecting progression.

Indicate which parameter you believe to be the most important in detecting progression.	Number of Responses
Anamnesis	0 (0.0%)
Myopic change	0 (0.0%)
Change in spherical equivalence	0 (0.0%)
Increased astigmatism	1 (5.3%)
Deteriorated best corrected visual acuity	0 (0.0%)
Deteriorated uncorrected visual acuity	0 (0.0%)
Corneal astigmatism	0 (0.0%)
Kmean	0 (0.0%)
Kmax	11 (57.9%)
MCT (minimum corneal thickness)	1 (5.3%)
Posterior radius (r-min)	0 (0.0%)
ISV (index of surface variance)	0 (0.0%)
D-index	0 (0.0%)
RPI (Pachymetry index)	0 (0.0%)
ART (Ambrósio Relation Thickness)	0 (0.0%)
KI (Keratoconus index)	0 (0.0%)
KPI (Keratoconus progression index)	0 (0.0%)
IHA (Index for height asymmetry)	0 (0.0%)
"DUCK SCORE"	0 (0.0%)
Belin ABCD progression display	4 (21.1%)
Other, please specify	2 (10.5%)
Total	19 (100.0%)

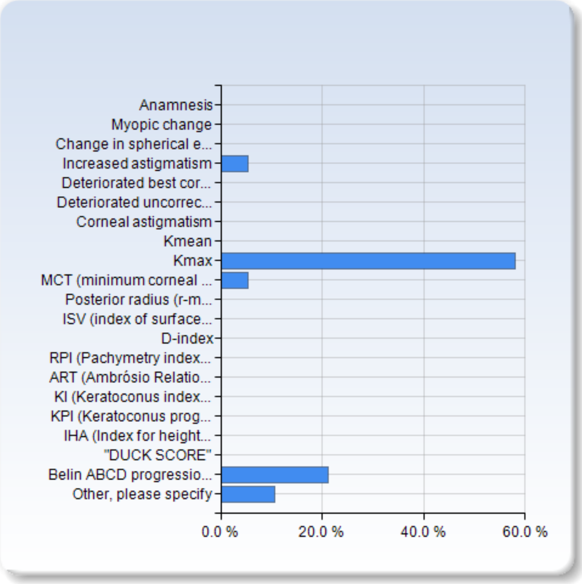

	Mean	Standard Deviation
Indicate which parameter you believe to be the most important in detecting progression.	12.4	5.7

Measurements by topography/tomography are usually compared between clinical visits to assess progression. Do you compare 1 measurement on one occasion against 1 measurement on another occasion, or do you calculate a mean of two or more measurements on one occasion and compare this mean against a mean of two or more measurements on another occasion?

Measurements by topography/tomography are usually compared between clinical visits to assess progression. Do you compare 1 measurement on one occasion against 1 measurement on another occasion, or do you calculate a mean of two or more measurements on one occasion and compare this mean against a mean of two or more measurements on another occasion?	Number of Responses
We use one (1) measurement on each occasion	11 (57.9%)
We use a mean of two or more measurements on each occasion	8 (42.1%)
Total	19 (100.0%)

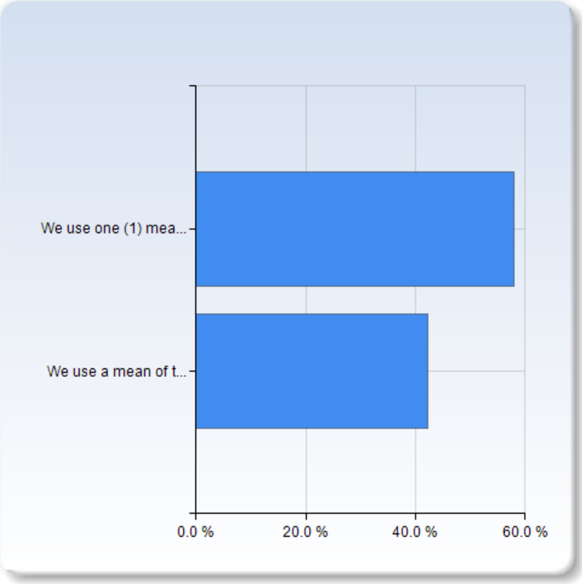

	Mean	Standard Deviation
Measurements by topography/tomography are usually compared between clinical visits to assess progression. Do you compare 1 measurement on one occasion against 1 measurement on another occasion, or do you calculate a mean of two or more measurements on one occasion and compare this mean against a mean of two or more measurements on another occasion?	1.4	0.5

Is there any subgroup in keratoconus in which it is more difficult to diagnose progression? If yes, please comment.

	Number of Responses
Subclinical keratoconus	2 (15.4%)
Moderate keratoconus	1 (7.7%)
Advanced keratoconus	12 (92.3%)
Total	15 (115.4%)

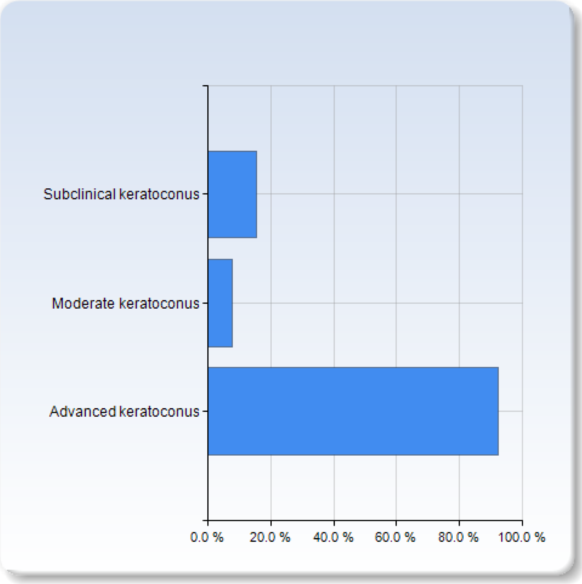

	Mean	Standard Deviation
	2.7	0.7

What technical system do you use to define progression? If you use more than one system, please specify when you use which system.

	Number of Responses
Scheimpflug-based? (Pentacam HR)	17 (89.5%)
Placido-based? (Orbscan)	3 (15.8%)
Scheimpflug with Placido? (Galilei)	0 (0.0%)
Other, please specify	4 (21.1%)
Total	24 (126.3%)

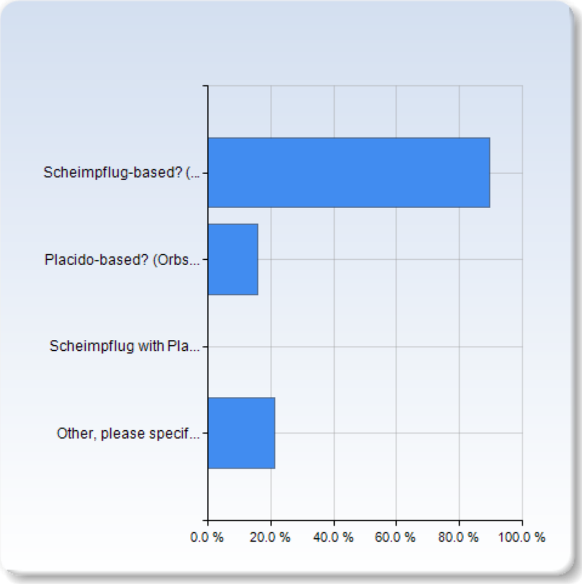

	Mean	Standard Deviation
	1.6	1.1

What power (W/cm2) do you use? If you use different powers for different patients, please specify why.

What power (W/cm2) do you use? If you use different powers for different patients, please specify why.	Number of Responses
3mW/cm2=30 minutes of UVA	8 (42.1%)
9mW/cm2=10 minutes of UVA	12 (63.2%)
18mw/cm2=5 minutes of UVA	3 (15.8%)
Other, please specify	1 (5.3%)
Total	24 (126.3%)

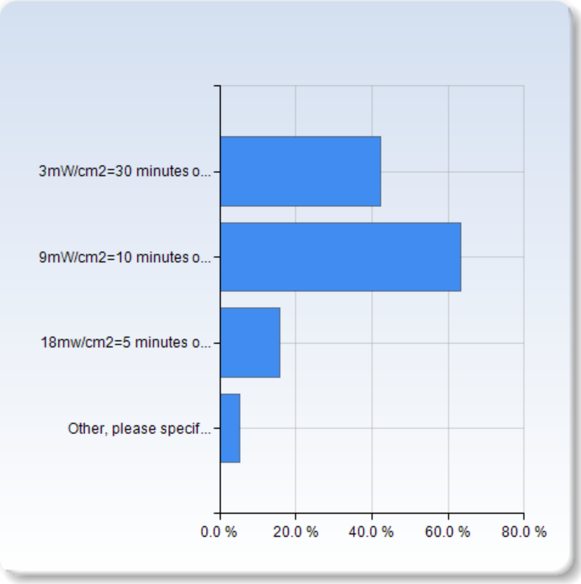

	Mean	Standard Deviation
What power (W/cm2) do you use? If you use different powers for different patients, please specify why.	5.6	2.4

Do you use pulsed or continuous UVA irradiation?

Do you use pulsed or continuous UVA irradiation?	Number of Responses
Continuous	16 (84.2%)
Pulsed	2 (10.5%)
Both types, please specify	1 (5.3%)
Other, please specify	0 (0.0%)
Total	19 (100.0%)

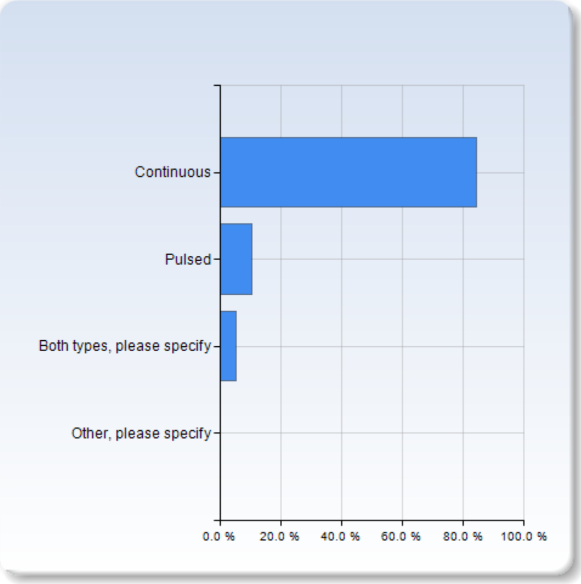

	Mean	Standard Deviation
Do you use pulsed or continuous UVA irradiation?	1.2	0.5

Which CXL protocols do you use clinically? If you use more than one technique, please specify why

Epi-on (No epithelial removal)

Epi-on (**No** epithelial removal)	Number of Responses
	4 (100.0%)
Total	4 (100.0%)

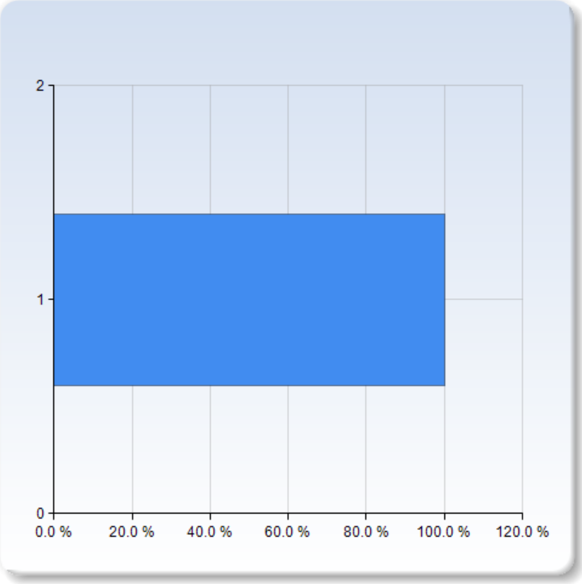

	Mean	Standard Deviation
Epi-on (**No** epithelial removal)	1.0	0.0

Epi-off (epithelial removal=classical protocol)

Epi-off (epithelial removal=classical protocol)	Number of Responses
	19 (100.0%)
Total	19 (100.0%)

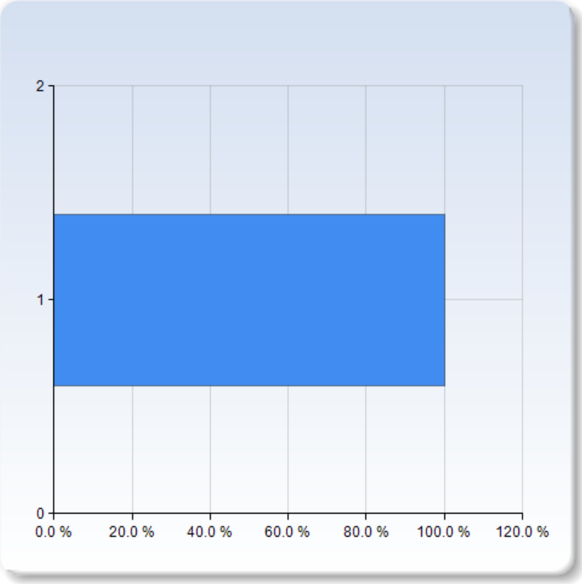

	Mean	Standard Deviation
Epi-off (epithelial removal=classical protocol)	1.0	0.0

Iontophoresis

Iontophoresis	Number of Responses
	0 (0.0%)
Total	0 (0.0%)

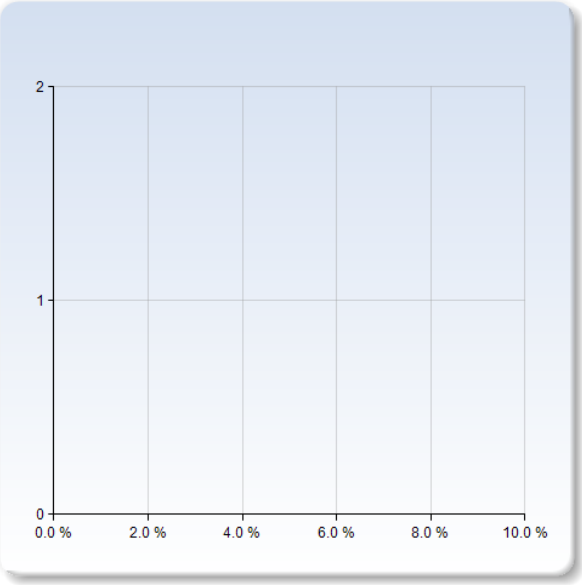

	Mean	Standard Deviation
Iontophoresis	0.0	0.0

Other, please specify

Other, please specify	Number of Responses
	3 (100.0%)
Total	3 (100.0%)

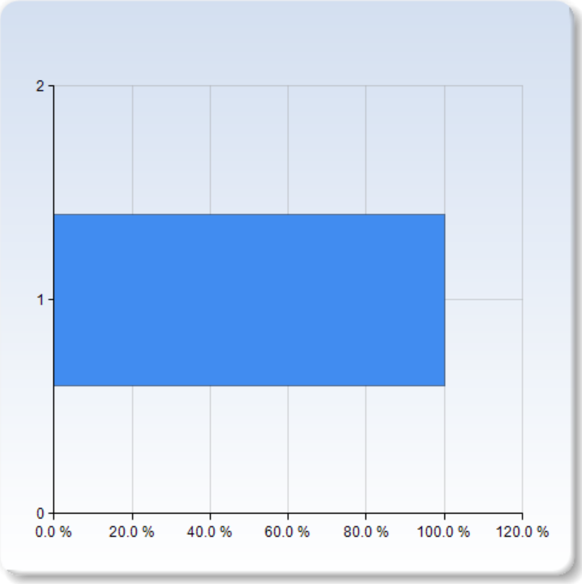

	Mean	Standard Deviation
Other, please specify	1.0	0.0

What kind of riboflavin do you use for epi-off techniques? If you use more than one type, please specify the indication for each type.

What kind of riboflavin do you use for epi-off techniques? If you use more than one type, please specify the indication for each type.	Number of Responses
Isotonic (with dextran)	7 (38.9%)
Hypotonic (without dextran)	10 (55.6%)
HPMC-based	8 (44.4%)
Other, please specify	0 (0.0%)
Total	25 (138.9%)

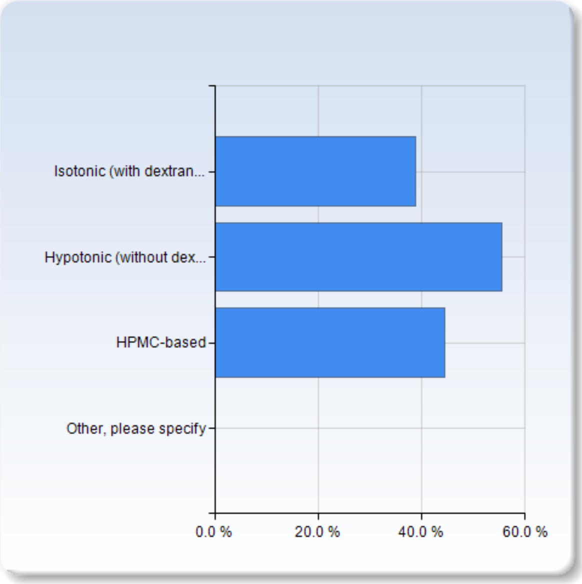

	Mean	Standard Deviation
What kind of riboflavin do you use for epi-off techniques? If you use more than one type, please specify the indication for each type.	2.0	0.8

Do you measure corneal thickness after epithelial debridement?

Do you measure corneal thickness after epithelial debridement?	Number of Responses
Yes	11 (57.9%)
No	8 (42.1%)
Total	19 (100.0%)

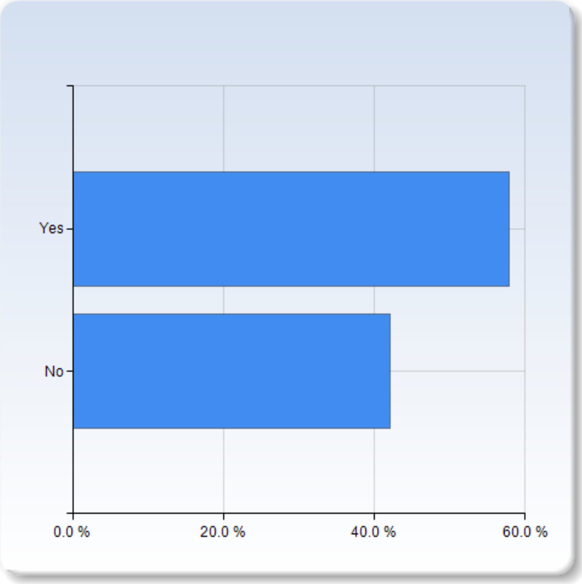

	Mean	Standard Deviation
Do you measure corneal thickness after epithelial debridement?	1.4	0.5

Do you measure corneal thickness immediately prior to UVA illumination?

Do you measure corneal thickness immediately prior to UVA illumination?	Number of Responses
Yes	12 (63.2%)
No	7 (36.8%)
Total	19 (100.0%)

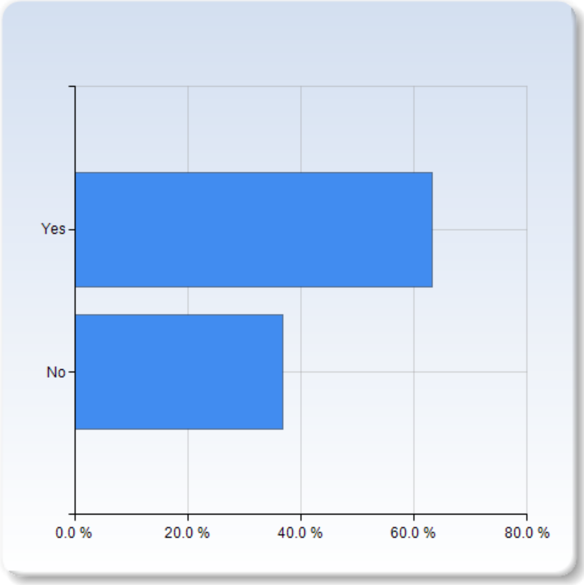

	Mean	Standard Deviation
Do you measure corneal thickness immediately prior to UVA illumination?	1.4	0.5

Do you check corneal thickness by repeated pachymetry during UVA irradiation?

Do you check corneal thickness by repeated pachymetry during UVA irradiation?	Number of Responses
Yes	3 (15.8%)
No	16 (84.2%)
Total	19 (100.0%)

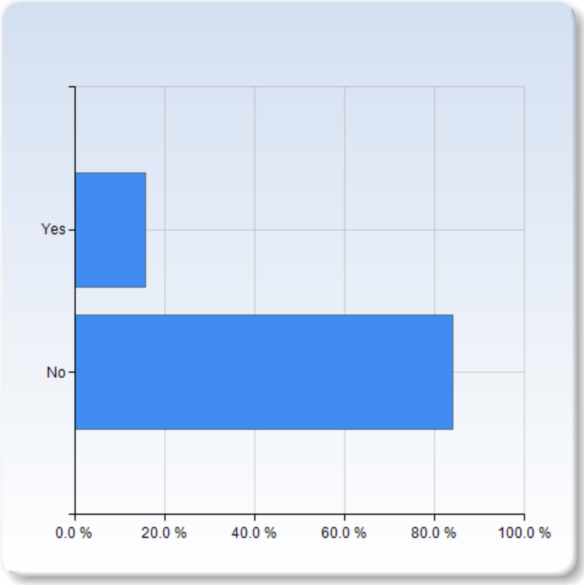

	Mean	Standard Deviation
Do you check corneal thickness by repeated pachymetry during UVA irradiation?	1.8	0.4

If the cornea is too thin to be treated safely which is your preferred approach?

If the cornea is too thin to be treated safely which is your preferred approach?	Number of Responses
Adding hypotonic riboflavin (=without dextran)	8 (42.1%)
Adding BSS on the cornea	0 (0.0%)
Adding sterile water	6 (31.6%)
Converting to contact lens-assisted CXL	0 (0.0%)
Other, please specify	5 (26.3%)
Total	19 (100.0%)

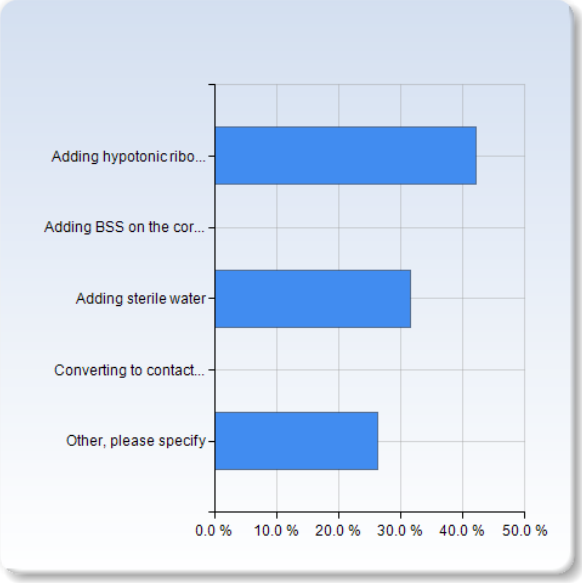

	Mean	Standard Deviation
If the cornea is too thin to be treated safely which is your preferred approach?	2.7	1.7

Have you had to abandon a CXL procedure due to insufficient corneal thickness?

Have you had to abandon a CXL procedure due to insufficient corneal thickness?	Number of Responses
Yes	10 (52.6%)
No	9 (47.4%)
Total	19 (100.0%)

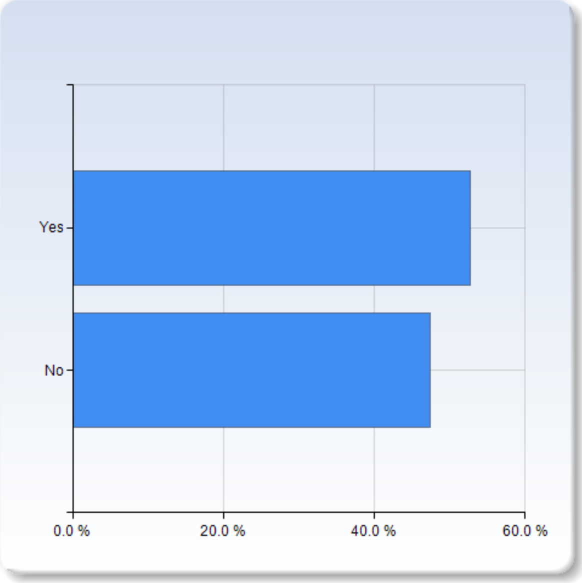

	Mean	Standard Deviation
Have you had to abandon a CXL procedure due to insufficient corneal thickness?	1.5	0.5

Do you add a soft contact lens after treatment?

Do you add a soft contact lens after treatment?	Number of Responses
Yes	9 (47.4%)
No	10 (52.6%)
Total	19 (100.0%)

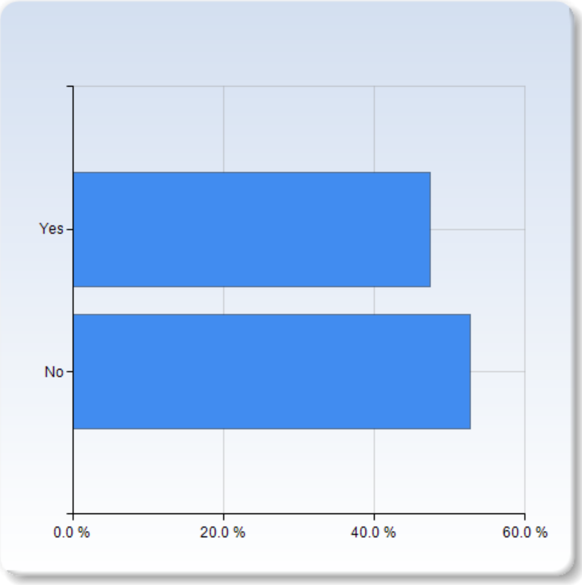

	Mean	Standard Deviation
Do you add a soft contact lens after treatment?	1.5	0.5

What is your standard pharmacological treatment after CXL?

What is your standard pharmacological treatment after CXL?	Number of Responses
Antibiotics	19 (100.0%)
Steroids	13 (68.4%)
NSAIDs	4 (21.1%)
Topical cycloplegic	5 (26.3%)
Topical anaesthetics	9 (47.4%)
Oral analgesics	17 (89.5%)
None	0 (0.0%)
Other, please specify	2 (10.5%)
Total	69 (363.2%)

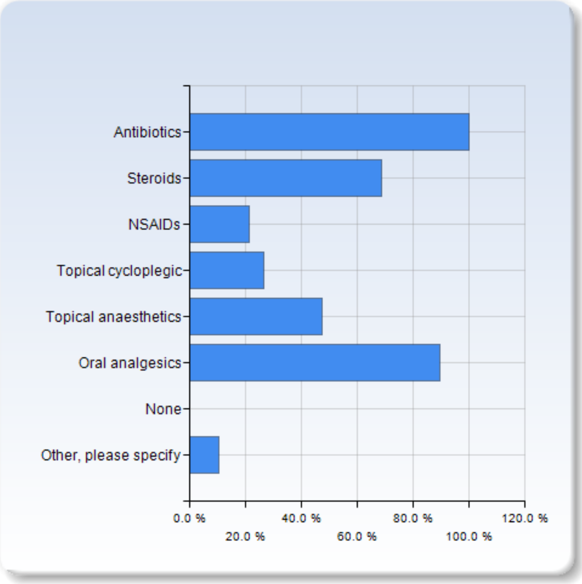

	Mean	Standard Deviation
What is your standard pharmacological treatment after CXL?	3.4	2.1

In a retrospective review, have you observed complications of CXL treatment for keratoconus?

In a retrospective review, have you observed complications of CXL treatment for keratoconus?	Number of Responses
Microbial infection	11 (64.7%)
Corneal melting	5 (29.4%)
Haze warranting treatment	5 (29.4%)
Re-activation of corneal herpes	1 (5.9%)
Delayed epithelial healing	14 (82.4%)
Other, please specify	3 (17.6%)
Total	39 (229.4%)

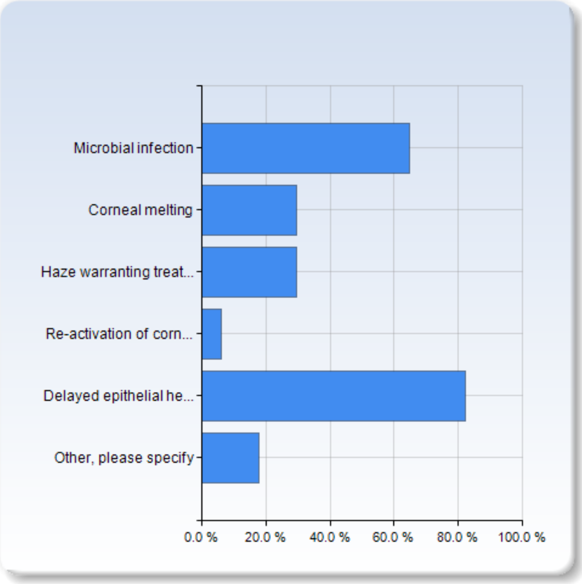

	Mean	Standard Deviation
In a retrospective review, have you observed complications of CXL treatment for keratoconus?	3.3	1.8

Do you use CXL for treatment of diseases other than keratoconus?

Do you use CXL for treatment of diseases other than keratoconus?	Number of Responses
Bullous keratopathy	2 (12.5%)
Infectious keratitis	15 (93.8%)
Other, please specify	4 (25.0%)
Total	21 (131.3%)

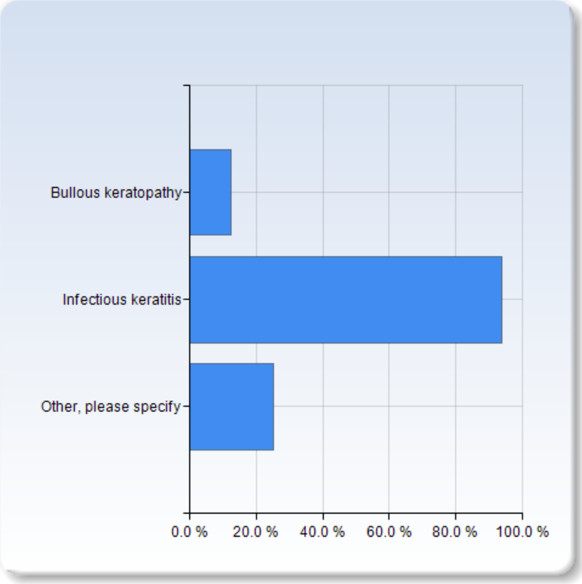

	Mean	Standard Deviation
Do you use CXL for treatment of diseases other than keratoconus?	2.1	0.5

Do you follow up patients after CXL?

Do you follow up patients after CXL?	Number of Responses
Yes	19 (100.0%)
No	0 (0.0%)
Total	19 (100.0%)

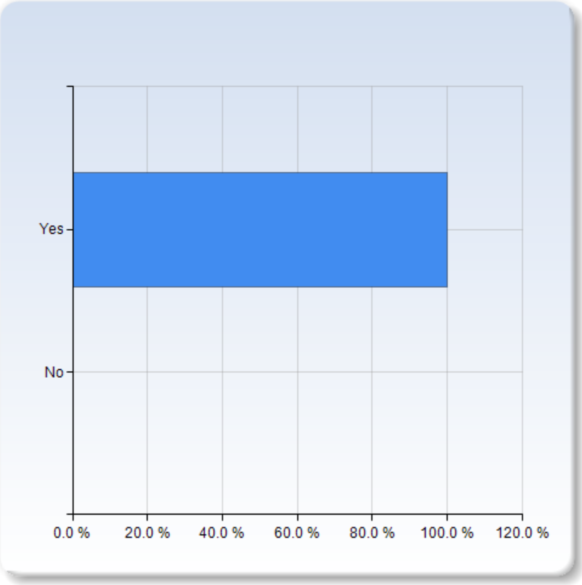

	Mean	Standard Deviation
Do you follow up patients after CXL?	1.0	0.0

Have you experienced a need for re-treatment with CXL?

Have you experienced a need for re-treatment with CXL?	Number of Responses
Yes	13 (72.2%)
No	5 (27.8%)
Total	18 (100.0%)

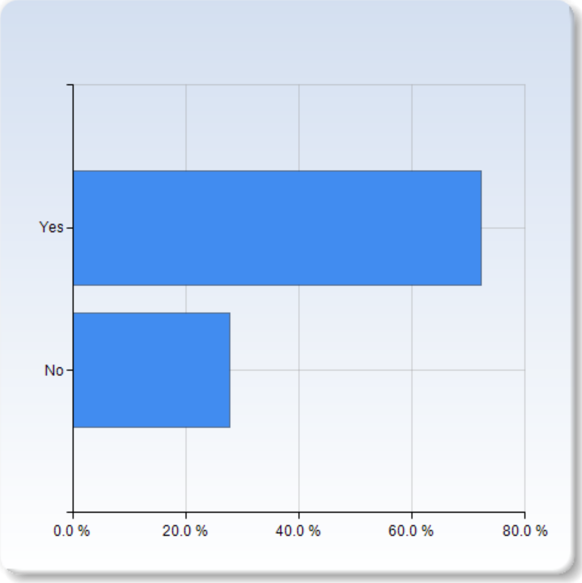

	Mean	Standard Deviation
Have you experienced a need for re-treatment with CXL?	1.3	0.5

What is your approach if there is progression after CXL treatment?

What is your approach if there is progression after CXL treatment?	Number of Responses
Re-treatment with the **same** CXL protocol	12 (63.2%)
Re-treatment with **different** CXL protocol (Please specify)	5 (26.3%)
Other, please specify	2 (10.5%)
Total	19 (100.0%)

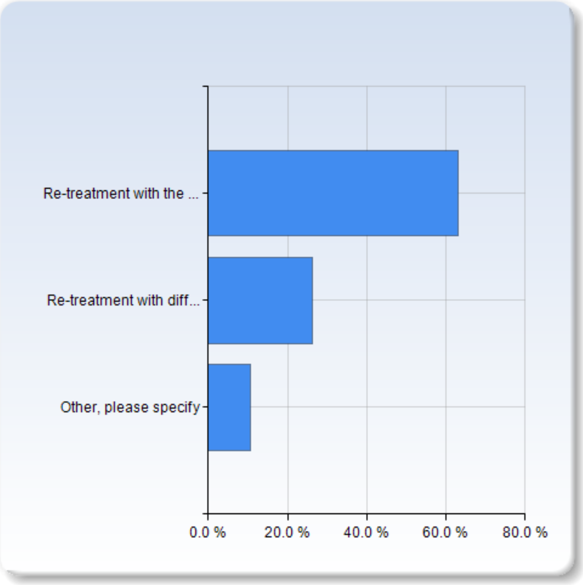

	Mean	Standard Deviation
What is your approach if there is progression after CXL treatment?	1.5	0.7

How do you evaluate the need for re-treatment?

How do you evaluate the need for re-treatment?	Number of Responses
Same as for untreated patients	18 (94.7%)
Other, please specify	1 (5.3%)
Total	19 (100.0%)

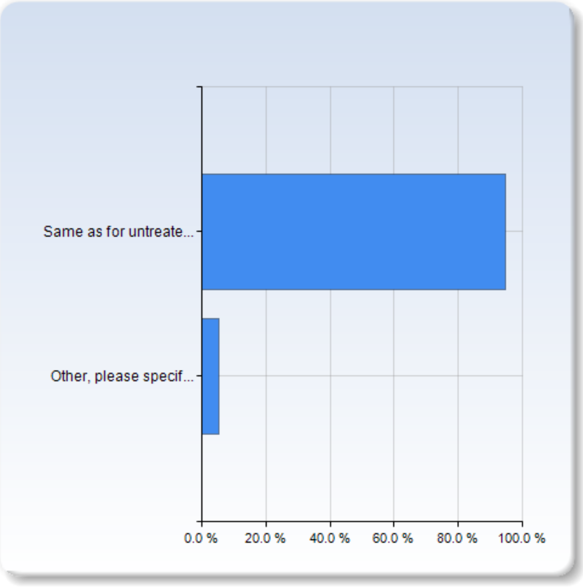

	Mean	Standard Deviation
How do you evaluate the need for re-treatment?	1.1	0.2

Which of the following aspects is in greatest need of improvement in CXL?

Which of the following aspects is in greatest need of improvement in CXL?	Number of Responses
Diagnosis	0 (0.0%)
How progression is defined	2 (10.5%)
Treatment protocol adaptation to different patients	5 (26.3%)
Development of effective epi-on protocols	4 (21.1%)
How to perform CXL in thin corneas	4 (21.1%)
Pain management	1 (5.3%)
How to avoid side-effects	0 (0.0%)
How to diagnose the need for re-treatment	2 (10.5%)
Other, please specify	1 (5.3%)
Total	19 (100.0%)

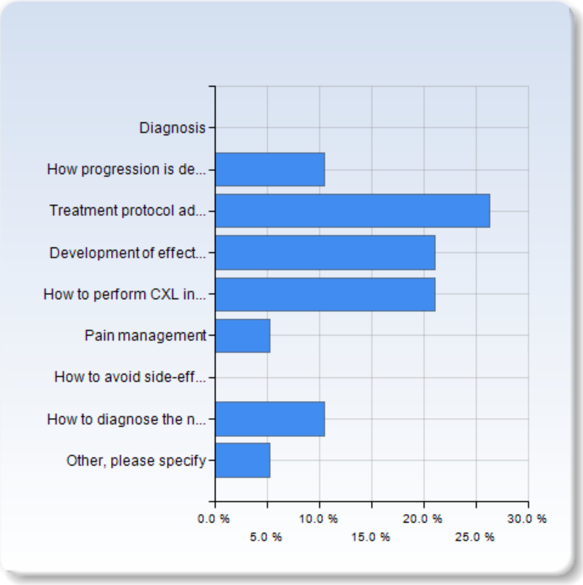

	Mean	Standard Deviation
Which of the following aspects is in greatest need of improvement in CXL?	5.2	2.6
